# The catalytic effect of calcium and potassium on CO_2_ gasification of Shengli lignite: the role of carboxyl

**DOI:** 10.1098/rsos.180717

**Published:** 2018-09-26

**Authors:** Yanpeng Ban, Yan Wang, Na Li, Runxia He, Keduan Zhi, Quansheng Liu

**Affiliations:** College of Chemical Engineering, Inner Mongolia University of Technology, Inner Mongolia Key Laboratory of High-Value Functional Utilization of Low Rank Carbon Resources, Huhhot 010051, Inner Mongolia, China

**Keywords:** Shengli lignite, calcium, potassium, CO_2_ gasification, catalysis mechanism

## Abstract

The CO_2_ gasification of Chinese Shengli lignite (SL) catalysed by K^+^ and Ca^2+^ was studied. The results showed that calcium could greatly decrease the gasification reaction temperature of SL, and the gasification reaction rates of acid-treated SL catalysed by calcium were significantly higher than that catalysed by potassium. Kinetic analysis showed that the activation energy of the reaction catalysed by calcium was much lower than that catalysed by potassium, which was the reason for the higher catalytic activity of calcium. Fourier transform infrared characterization showed that, compared with acid-treated SL, the addition of K^+^/Ca^2+^ resulted in the significant weakening of C=O bond, and new peaks attributed to carboxylate species appeared. X-ray photoelectron spectroscopy results indicated that the numbers of C=O decreased after the metal ions were added, indicating the formation of metal–carboxylate complexes. Raman characterization showed that the *I*_D1_/*I*_G_ values increased, suggesting more structural defects, which indicated that the reactivity of coal samples had a close relation with amorphous carbon structures. Ca^2+^ could interact with the carboxyl structure in lignite by both ionic forces and polycarboxylic coordination, while K^+^ interacted with carboxyl structure mainly via ionic forces.

## 1. Introduction

Highly efficient and clean utilization of the low rank coals (LRCs) has attracted wide attention around the world due to the large reserves of LRCs. Catalytic gasification is an important way for the utilization of LRCs. Gasification is a thermal chemical process by which the solid fuels are converted to combustible gases in the presence of different gas atmosphere, including steam, oxygen or carbon dioxide. The coal gasification can be divided into three main steps: pyrolysis, volatile matter reforming, and char gasification, and the last step is considered to be the rate-controlling step because it is kinetically slower compared with the other two steps [1,2]. The pyrolysis and devolatilization steps mainly produce tars and release no significant gases, such as CO, CO_2_, H_2_ and CH_4_ in the temperature range of 300–500°C. The gasification process takes place at least above 500°C. Although the devolatilization process takes place at the first stage, the volatiles may not be completely removed during the gasification process especially when the gasification agents are insufficient [3,4]. In the gasifier, the devolatilization and gasification take place simultaneously. The major reactions involved in the gasification process include combustion (reaction with O_2_), Boudouard reaction (reaction with CO_2_), and steam gasification (reaction with steam) [5]. Char is formed simultaneously with the coal gasification reaction. The reaction between char and H_2_O or CO_2_ is the rate-determining step. Comparatively, the reactivity of CO_2_ with char is much less than that with steam, especially at temperatures lower than 900°C. So Boudouard reaction is generally considered as the rate-determining step in the gasification process [6]. Increasing the Boudouard reaction rate below 900°C is the key step for low-temperature gasification, which can decrease the energy consumption of the gasification process [7].

Catalytic gasification is one of the most attractive options to achieve low-temperature gasification due to the high gasification reaction rate, the selectivity in reaction pathways, and the useful gas products [8]. Moreover, the costs associated with the gasification and the requirements for the related facilities can be reduced by the catalytic approach [9]. Coal contains alkali metals, alkaline earth metals, and transition metals, such as Na, K, Ca, Mg and Fe [10–13]. The contents of some of these elements within coals are often lower than 1 wt%, but they present a significant catalytic effect on coal gasification as well as obvious influences on the structures of coals during the gasification process. Alkali and alkaline earth metals were reported to be efficient in promoting CO_2_ gasification of coal, and thus adding metal salts as additives during CO_2_ gasification of coal has served as a hot topic in the field of coal conversion [14–16]. Potassium has been reported extensively as an effective catalyst for coal gasification [17–19]. Calcium also exhibits considerable catalytic effect on coal gasification [20]. The catalytic effects of potassium and calcium on coal gasification were studied extensively. However, the results about the catalytic effects of potassium and calcium obtained by different researchers were still inconsistent because of the variation in composition and properties of different coals [21]. To the best of our knowledge, most researchers reported that the catalytic effects of potassium on the CO_2_ gasification of coal or char are much more remarkable than those of calcium [22–26]. Only few researchers reported that the catalytic effect of calcium was superior to that of potassium on coal gasification [27,28]. The catalytic gasification of coal using calcium as the catalyst has several advantages over potassium, such as lower costs and corrosiveness, abundant reserves on earth, and facile accessibility.

Recently, our previous studies showed that the catalytic effects of calcium were superior to those of potassium on the gasification of Chinese Shengli lignite (SL) [29,30]. In this work, the interaction between lignite and K^+^ or Ca^2+^ is to be investigated, and the possible mechanisms of the catalysing gasification reaction are attempted to be studied.

## 2. Experimental

### 2.1. Sample preparation

Lignite obtained from Shengli coalfield in Inner Mongolia of China was used as the raw material. The received SL samples contained 47.6% moisture, 7.70% ash, 17.3% volatiles and 27.4% fixed carbons. The sample was ground, sieved to the size fraction of 38–74 µm for further processing, and dried in nitrogen atmosphere at 105°C for 4 h.

The demineralized SL sample was obtained by leaching with hydrochloric acid. Approximately 100 g of SL was mixed with 1000 ml 18% hydrochloric acid aqueous solution and stirred for 12 h at room temperature. The resulting slurry was filtered by a vacuum filter, rinsed repeatedly with distilled water to remove the residue chloride ions, and dried in nitrogen atmosphere at 105°C for 4 h, designated as SL^+^. The solution was configured as the ratio of 1 g SL^+^ and 3 ml acid solution of HF (greater than 40%) and soaked for 12 h. The suspension was filtered and washed with distilled water until pH neutrality and then dried at 105°C for 4 h, denoted as SL^++^. The dried SL and SL^++^ were also prepared similar to SL^+^. According to the previous results [31], the proximate and ultimate analyses of SL, SL^+^ and SL^++^ are listed in table 1. Demineralized coal samples and mass fraction of the main metal elements are presented in table 2.

**Table 1.** Proximate and ultimate analysis of coal samples.

	proximate analysis (wt%)				ultimate analysis (wt%)				
sample	M_ad_	A_d_	V_d_	F_cd_	C	H	N	S	O^f^
SL	1.52	13.92	33.37	52.71	57.59	3.58	0.89	1.81	22.21
SL^+^	2.14	7.53	39.77	52.70	61.42	3.34	0.86	1.74	25.10
SL^++^	2.18	1.14	41.97	56.89	64.90	4.88	0.91	1.69	26.48

**Table 2.** Metal ion percentage in coal samples and ashes.

	coal based/ash based (wt%)						
sample	Al^3+^	Na^+^	Ca^2+^	Si^4+^	Fe^n+^	K^+^	Mn^n+^
SL	2.40/35.50	0.58/8.59	0.41/6.08	3.11/46.09	0.11/1.69	0.08/1.18	0.06/0.88
SL^+^	0.90/32.94	0.00/0.16	0.01/0.19	2.89/64.58	0.03/0.92	0.03/1.18	0.00/0.03
SL^++^	0.11/41.77	0.00/1.03	0.01/2.10	0.14/53.31	0.00/1.22	0.00/0.13	0.00/0.45

The reactivity of the gasification of SL^+^ and SL^++^ is similar [32]. Therefore, the metal-cation-doped SL^+^ coal samples were used to investigate the catalytic effect of different metals during CO_2_ gasification. The dosages of all metal ions were 5 wt% of total coal sample. KOH and Ca(OH)_2_ were added to SL^+^ by the impregnation method, named as SL^+^-K and SL^+^-Ca.

### 2.2. CO_2_ gasification

**Figure 1.**
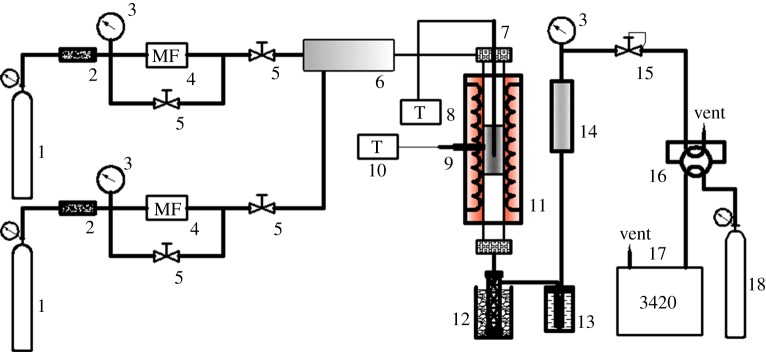
Flow chart of the fixed-bed reactor for CO_2_ gasification reactivity test of coal samples. 1. Reactant gas; 2. Filter; 3. Pressure gauge; 4. Mass flow meter; 5. Pressure reducing valve; 6. Mixer; 7. Temperature thermocouple; 8. Thermograph; 9. Temperature control thermocouple; 10. Temperature controller; 11. Reactor; 12. Ice-bath; 13. Cold hydrazine; 14. Purifier; 15. Back pressure valve; 16. Six-port valve; 17. Chromatograph; 18. Carrier gas.

The CO_2_ gasification reaction was evaluated in a CCG-2010 fixed-bed quartz reactor. Figure 1 shows a schematic of the experimental set-up for CO_2_ gasification.

The reaction system consists of a steam generator and a fixed-bed reactor that is made of stainless steel (8 × 350 mm). The reactor is heated by an electric furnace. For each experiment, 150 mg of coal sample was loaded in the central zone of the tubular reactor sandwiched by quartz wool and quartz sand in order to maintain homogeneous temperature. Pressure of the reactor was adjusted to 0.25 MPa by reactant gas (Ar+9%CO_2_) at a flow rate of 110 ml min^−1^. The reactor was heated up from room temperature to 600°C at the heating rate of 15°C min^−1^, and up to 1000°C at 2°C min^−1^.

The produced gas passed through an ice–water trapper followed by a cooler maintained at −30°C to prevent steam and tar matters from entering into the gas chromatography analyser. The concentrations of H_2_, CO, CO_2_ and CH_4_ in the off gas were quantitatively analysed by an online gas chromatograph (3420) equipped with a thermal conductivity detector, and the sampling interval was 4 min.

The gas instantaneous release rates (*F_i_*) of H_2_, CO, CO_2_ and CH_4_ are given by

2.1 $$F_{i}=\frac{((110\times y_{i})/(100-\sum y_{i}))\times 100}{22.4\times m_{0}}\;\;\;(i={\text{H}}_{2},\;\;\text{CO},\;\;\text{C}{\text{H}}_{4}\;\text{and}\;\text{C}{\text{O}}_{2}),$$

where $\;y_{H_{2}},\;\;y_{\mathrm{CO}},\;\;y_{CH_{4}},\;\;y_{CO_{2}}\;\text{and}\;y_{\mathrm{Ar}}$ are the concentrations (v/v%) of H_2_, CO, CH_4_, CO_2_ and Ar respectively, and *m*_0_ (g) is the original carbon mass.

The carbon conversion (*X*) and gasification rate (*r*) were calculated using the following equations:

2.2 $$X=\frac{\int_{t_{0}}^{t}F_{i}\times \text{d}t}{\int_{t_{0}}^{t_{g}}F_{i}\times \text{d}t}\times 100\text{\%}\;\;\;(i=\text{CO})$$

and

2.3 $$r=\frac{\text{d}x}{\text{d}t},$$

where *X* is the conversion of the coal samples, *t*_0_ the start of gasification (min), *t*_g_ the termination of gasification (min), *t* the reaction time (min) [33].

The catalytic efficiency index (EI) is also indicative of the catalytic performance. The EI of the doped metal is defined as follows:

2.4 $$\text{EI}=\frac{r_{SL^{+}\;-\;\;\mathrm{Doped}}-r_{SL^{+}}}{r_{SL^{+}}}.$$

### 2.3. Characterization methods

The Fourier transform infrared (FTIR) spectra of the samples were recorded in the range of 400–4000 cm^−1^ by a Nicolet FTIR spectrometer NEXUS670, and the spectral resolution was 4 cm^−1^. The potassium bromide and coal samples were dried in an oven at 105°C for 4 h before the FTIR investigation. Through the KBr pellet technique, coal samples and KBr at 1 : 200 were ground together to a fine powder and mixed well.

Raman spectroscopy with a 532 nm laser was used to investigate chemical structural changes of different samples. Raman analysis was conducted at room temperature by a Thermo Fisher spectrometer equipped with a DXR detector. The spectra were collected in the range of 500–3500 cm^−1^. Raman analysis was performed by the method of baseline drift correction and Gaussian peak-differentiating fitting.

Surface composition of the samples was determined by X-ray photoelectron spectroscopy (XPS) with Al K-α radiation using a PHI-5400 spectrometer from America. The analysis area was 300 × 300 µm. The degree of vacuum was 3 × 10^−7^ Pa. The calibration was performed using the main C1s peak at 284.6 eV.

## 3. Results and discussion

### 3.1. CO_2_ gasification reactivity

**Figure 2.**
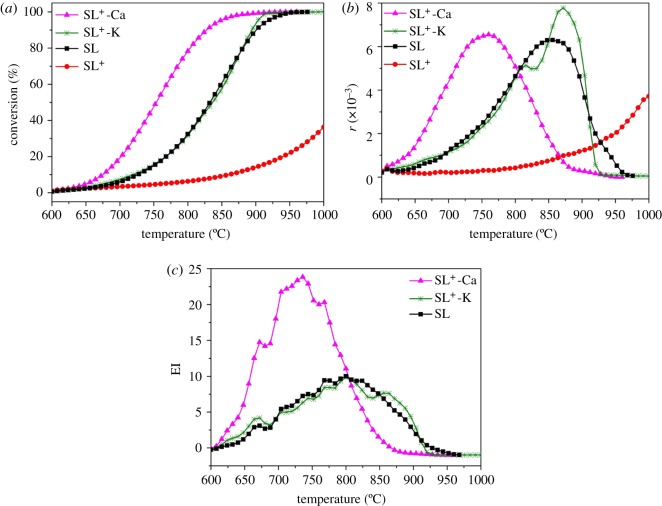
CO_2_ gasification performance of Shengli lignite. (*a*) CO_2_ gasification conversion, (*b*) reactivity and (*c*) catalytic efficiency index (EI).

The CO_2_ gasification was performed in a fixed-bed reactor system. Figure 2*a* demonstrates that the carbon conversion of CO_2_ gasification reaction was enhanced with the increase of the temperature for all lignite samples. The rate of CO_2_ gasification reaction of each sample was very low and could be negligible at temperatures lower than 700°C. The CO_2_ gasification conversion of SL^+^ increased to 15% at 900°C, which was much lower than those of SL^+^-Ca and SL^+^-K. SL^+^-Ca was converted almost completely at 850°C, and the conversions of SL^+^-K and SL^+^ were 60% and 10%, respectively.

The carbon conversion rates of CO_2_ gasification are shown in figure 2*b*. The reactivity of the SL^+^ sample increased slowly with the increasing of the temperature from 600 to 1000°C, but the reactivity of the SL^+^-Ca and SL^+^-K samples increased much faster. The CO_2_ gasification reactivity and reaction rate of SL^+^-Ca increased significantly once the temperature was higher than 600°C and reached the maximum at 750°C. Relatively, the CO_2_ gasification reaction rate of SL^+^-K increased steadily from 600 to 760°C. The doped Ca^2+^ and K^+^ showed obvious catalytic effects on CO_2_ gasification of SL^+^, and the catalytic effect of Ca^2+^ was more obvious.

EI is used as a useful parameter for evaluating the catalytic efficiency of doped metal for the CO_2_ gasification of lignite samples. It can be seen from figure 2*c* that the EI of SL^+^-Ca was extremely high from 650 to 800°C. The EI of SL^+^-K is relatively much lower at the same temperature range.

The above results indicated that calcium exhibited significant catalytic effect on the CO_2_ gasification reaction of Shengli lignite. Calcium greatly improved the reactivity and reduced the reaction temperature windows. The added potassium had similar catalytic effect on the CO_2_ gasification reaction of SL^+^ with the inherent minerals. The catalytic effect of calcium on CO_2_ gasification reaction is superior to that of potassium.

### 3.2. Kinetic analyses of CO_2_ gasification

The rate of the gas–solid reaction in a chemical reaction can generally be expressed by the following equation (3.1) [34]:

3.1 $$\frac{\text{d}x}{\text{d}t}=k\cdot {\left(1-x\right)}^{n},$$

where *k* is the reaction rate constant (min^−1^), and *n* is the reaction index. The Arrhenius equation can be expressed as follows:

3.2 $$k=A\;\exp\;\left[-\frac{E}{R\cdot (T+273.15)}\right]\;,$$

where *R* is a gas constant (8.314 J mol^−1^ K^−1^), *E* is the activation energy (kJ mol^−1^), *T* is the gasification temperature (K) and *A* is a frequency factor (min^−1^).

From equations (3.1) and (3.2), it can be concluded that:

3.3 $$\ln\;\left[\frac{\text{d}x}{\text{d}t}\frac{1}{{\left(1-x\right)}^{n}}\right]=\ln\;A-\frac{E_{a}}{RT}.$$

The activation energy *E*_a_ and frequency factor *A* can be calculated from the linear fitting of $\ln[(\text{d}x/dt)\;(1/{\left(1-x\right)}^{n})]\;\mathrm{versus}\;1/T$.

**Figure 3.**
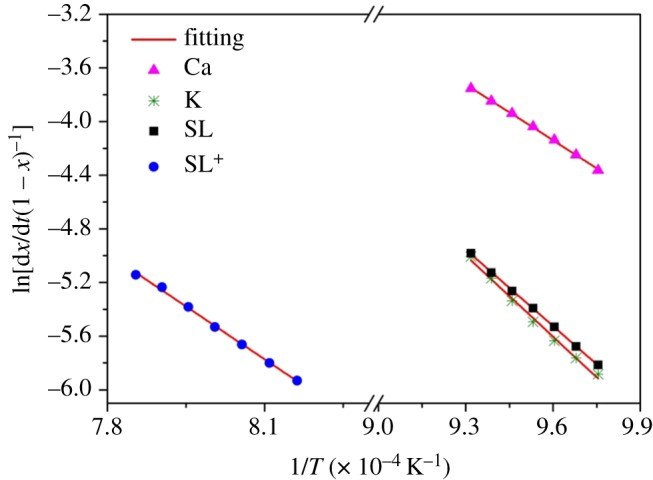
Linear fitting of ln[d*x*/d*t*(1 − *x*)] versus 1/*T* of samples.

The linear fittings of $\ln[(\text{d}x/dt)\;(1/{\left(1-x\right)}^{n})]\;\mathrm{versus}\;1/T$ of SL^+^-Ca, SL^+^-K, SL and SL^+^ corresponding to main reaction ranges are illustrated in figure 3. The values of frequency factor *A*, activation energy *E*_a_ and linear correlation coefficients *R*^2^ of SL^+^-Ca, SL^+^-K, SL and SL^+^ are listed in table 3. The CO_2_ gasification activation energies of SL^+^-Ca, SL^+^-K, SL and SL^+^ were 115.6, 167.8, 157.9 and 218.4 kJ mol^−1^, respectively. The addition of calcium reduces the activation energy of CO_2_ gasification reaction by 102.8 and 57.7 kJ mol^−1^ compared to SL^+^ and SL, respectively, which might contribute to the significant catalytic effect of calcium on the gasification reaction. The addition of potassium reduced the activation energy of CO_2_ gasification reaction by 50.6 kJ mol^−1^ compared to that of SL^+^ and slightly higher than that of SL. The activation energy of CO_2_ gasification reaction of the SL sample was lower than that of SL^+^, which proved the catalytic effect of inherent minerals on the CO_2_ gasification of Shengli lignite.

**Table 3.** Kinetic parameters of coal samples for CO_2_ gasification.

samples	temperature zone Δ*T* (°C)	*E*_a_ (kJ mol^−1^)	*A* (min^−1^)	*R*^2^
SL^+^-Ca	752–800	115.6	9.97 × 10^3^	0.9988
SL^+^-K	752–800	167.8	9.55 × 10^5^	0.9935
SL	752–800	157.9	3.31 × 10^5^	0.9997
SL^+^	952–1000	218.4	5.43 × 10^6^	0.9977

### 3.3. Characteristics of samples

#### 3.3.1. Fourier transform infrared spectroscopy

**Figure 4.**
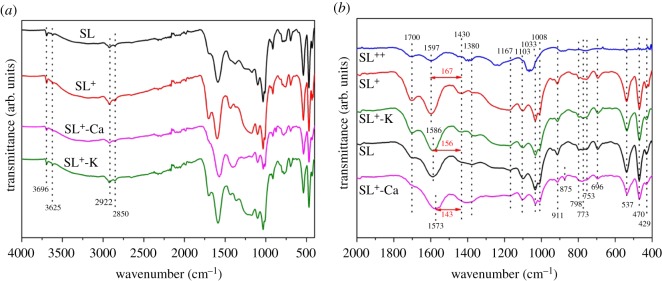
FTIR spectra of coal samples.

The interaction between calcium or potassium cations and the inherent oxygen functional group within the lignite samples was confirmed by infrared spectroscopy. The FTIR spectra of the four samples are compared in figure 4*a*.

All of the samples exhibit a broad transmission band at approximately 3420 cm^−1^, which could be attributed to the –OH stretching vibration, and bands at 2923 cm^−1^ and 2848 cm^−1^ were resolved into stretching vibrations of aliphatic –CH, –CH_2_, and –CH_3_. A typical 400–2000 cm^−1^ band in the FTIR spectrum is shown in figure 4*b*. It may be confirmed that the wavenumbers lower than 950 cm^−1^ were mainly attributed to the inherent mineral aluminium and silicon component in the SL^+^ by comparison with SL^++^. The most significant difference of infrared spectra lies in 1250–1750 cm^−1^. The band at 1597 cm^−1^ could be assigned to the stretching vibration of C–O bonds in a carboxyl structure, which should be between the bands of C–O and C=O bonds, and could not be clearly distinguished, because they were usually shielded by the oxygen containing functional groups in LRC varieties [35]. The most significant difference of infrared spectra lies in 1250–1750 cm^−1^. The FTIR analyses of SL and SL^+^ samples demonstrated reversible conversion of the metal carboxylate groups to carboxyl groups by the acid treatment. The peak intensities of SL^+^ and SL^++^ were increased at 1700 cm^−1^. The addition of Ca^2+^ and K^+^ into SL^+^ leads to the decrease of the band intensity at the same position because –COOH was transformed into –COOM in the presence of metal ions and the C=O absorption peak was weakened [36,37]. These results meant that the addition of Ca^2+^ and K^+^ resulted in significant changes of the original C=O due to the formation of metal–carboxylate complexes. The separation of the FTIR bands ($\Delta v=v_{\mathrm{as}}(\text{C}{\text{O}}_{2}^{-})-v_{s}(\text{C}{\text{O}}_{2}^{-}))$ was indicative of the structure of carboxylate [38]. The calculated Δv for SL^+^, SL^+^-K and SL^+^-Ca are 167, 156 and 143 cm^−1^, respectively. The addition of Ca^2+^ and K^+^ results in the decrease of the FTIR band separation Δv due to the metal–carboxylate interactions. It can be postulated that the combination of carboxyl functional groups with potassium in SL^+^-K is via ionic configurations because these configurations are very common for alkali metal carboxylates [39]. It is impossible to use the Δv value alone to differentiate either ionic or bridging configuration structures in SL^+^-Ca. It was reported that calcium acetate had two types of acetate groups, one of which displayed more covalent character and the other was purely ionic force [40]. So it could be deduced from FTIR band Δv values that the Ca^2+^ complexes in SL^+^-Ca existed via both covalent bonding and ionic configurations simultaneously [41].

#### 3.3.2. Raman spectroscopy

**Figure 5.**
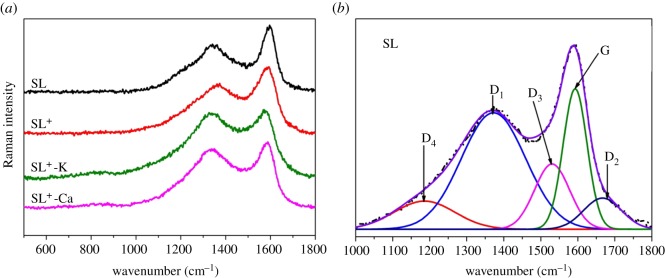
Raman spectra of (*a*) coal samples and (*b*) the fitted curves of SL.

Raman spectra were also used to investigate the structural changes after adding metal ions. Raman spectra of coal samples are shown in figure 5*a*. In order to get more detailed carbon skeleton structure of coal, each sample was fitted into five peaks according to the literature [42,43]. The fitting curves of Raman spectrum of SL are shown in figure 5*b*.

Table 4 shows the variation trend of the fitting parameters (*I*_D1_/*I*_G_ and *I*_G_/*I*_all_) with the different metal ion addition. *I*_D1_/*I*_G_ can be used to characterize the surface defect degree in coal samples. With the increase of *I*_D1_/*I*_G_, the graphitization was reduced. The ratio of *I*_G_/*I*_all_ reflects the amount of polyaromatic structure in coal sample. The metal ions combined with the carboxyl groups in the aromatic structure of coal, which affected the stability of polycyclic aromatic hydrocarbons to a certain extent. Therefore, the ratio of *I*_G_/*I*_all_ gradually decreased, and SL^+^ has the maximum degree of graphitization. The greater the degree of graphitization, the fewer the defects and the more stable the structure. It can be deduced that the amount of defects increased due to the introduction of the metal ions, and the contents of graphite crystal structures decreased compared with SL^+^. The reactivity of the SL^+^ samples was improved by the addition of metal ions. Therefore, it could be postulated that the reactivity of coal samples had a close relation with amorphous carbon structures.

**Table 4.** Raman parameters of the coal samples after adding different metal ions.

sample	*I*_G_/*I*_all_	*I*_D1_/*I*_G_	*L*_a_ (nm)
SL^+^-Ca	0.189	3.144	1.576
SL^+^-K	0.222	2.232	2.221
SL	0.220	2.235	2.300
SL^+^	0.227	2.174	2.280

#### 3.3.3. X-ray photoelectron spectroscopy

**Figure 6.**
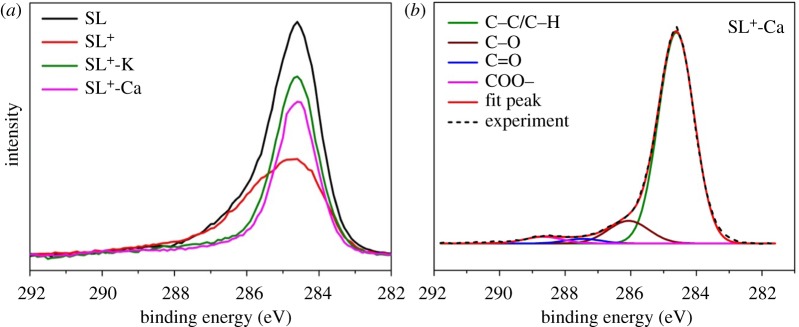
(*a*) XPS C1s spectra of coal samples and (*b*) the fitted curves of SL^+^-Ca.

The main C1s XPS spectrum was used to characterize the surface elemental composition of coals. Figure 6 shows XPS C1s spectra of coal samples and fitting chart diagram of SL^+^-Ca. The carbon in coal existed in four forms, as shown in table 5, according to the literature [44,45]. By comparing the C1s spectra of samples, it can be found that the proportion of C–C/C–H had no obvious change after the addition of calcium and potassium. The content of C–O was higher than that of SL^+^, which meant that adding calcium and potassium could lower the aromaticity. The content of C=O also decreased probably due to the formation of the –COOM structure after adding the metal ion, which was proved by the FTIR results. The increase of C–O and COO– might be mainly due to the addition of calcium and potassium cations. The combination of the metals and COO– functional group led to more disorder of the coal structure of the samples.

**Table 5.** The XPS C1s spectra data of the coal samples.

carbon form	position (eV)	SL^+^-Ca	SL^+^-K	SL	SL^+^
C–C/C–H	284.6	84.94	86.79	85.63	84.80
C–O	286.3	10.31	9.57	7.99	8.58
C=O	287.5	1.88	1.10	4.14	3.55
COO–	289.0	2.87	2.54	2.25	3.07

### 3.4. Possible interaction between K^+^ or Ca^2+^ and the carboxyl structure

**Figure 7.**
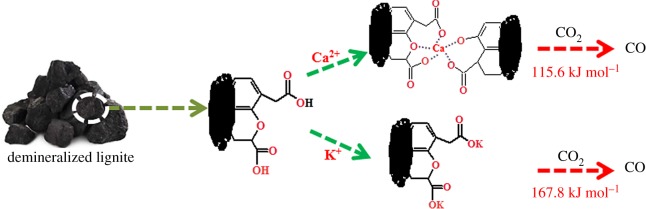
The possible interaction mode of K^+^ and Ca^2+^ with the carboxyl structure.

Lignite is well known to contain amounts of oxygen, and the basic structure of the coal unit includes the polycondensation of various aromatic rings [46,47], single aromatic ring or heterocyclic rings, and the connected hydroxyl (–OH), carboxyl (–COOH), carbonyl (–C=O), methoxy (–OCH_3_), as well as metal ions coordinated with oxygen functional group [48]. These basic structural units were connected by bridge bond to give rise to the macromolecular structure, and the cage type three-dimensional structure was formed by hydrogen bonds [49–54]. Based on the results in this work and the structure of the coal, the possible interaction between K^+^ or Ca^2+^ and the carboxyl structure is shown in figure 7. Demineralized coal sample contained a lot of oxygen-containing functional groups, such as carboxyl group and phenolic hydroxyl. The metal–carboxylate interactions formed when metal ions were added into demineralized coal samples. K^+^ interacted with the carboxyl structure in lignite in the form of ionic forces, while Ca^2+^ interacted with the lignite in the form of both ionic forces and polycarboxylic coordination. The different interaction forms led to different effects on the activation energy of the gasification reaction. The addition of K^+^ led to the decrease of the activation energy from 218.4 kJ mol^−1^ (SL^+^ sample) to 167.8 kJ mol^−1^, while the activation energy for Ca^2+^ added sample decreased to 115.6 kJ mol^−1^. It could be speculated that the lower activation energy for Ca^2+^ added samples contributed to the superior catalytic performance compared with K^+^ during CO_2_ gasification reaction.

## 4. Conclusion

In summary, the gasification reactivity and the structure changes of different SL samples during CO_2_ gasification process were investigated. It was proved that Ca^2+^ and K^+^ could significantly promote the CO_2_ gasification, and Ca^2+^ exhibited higher activity than potassium. Kinetic analysis indicated that the activation energies of SL^+^-Ca, SL^+^-K and SL^+^ samples were 115.6, 167.8 and 218.4 kJ mol^−1^, respectively. Detailed characterization showed that K^+^ interacted with the carboxyl structure in lignite mainly in the form of ionic forces, while Ca^2+^ interacted with the lignite in the form of both ionic forces and polycarboxylic coordination. It could be deduced that the different interaction forms of Ca^2+^ and K^+^ with the carboxyl structure in lignite led to the differences in activation energy, and finally resulted in the superior activity of Ca^2+^ compared with K^+^. It is believed that this result could be applied to all LRC with high oxygen functional group content, especially carboxyl structure.

## Supplementary Material

Repeatability and reproducibility，Supplementary Methods

## Data accessibility

Supporting data for this article have been uploaded as part of the electronic supplementary material.
