# Supplementary material for: The catalytic effect of calcium and potassium on CO2 gasification of Shengli lignite: the role of carboxyl
Source: R Soc Open Sci. 2018 Sep 26;5(9):180717. doi: 10.1098/rsos.180717 (PMC6170535; doi:10.1098/rsos.180717)
Supplement: Repeatability and reproducibility，Supplementary Methods [file rsos180717supp1.docx]

**Supporting Material**

**The catalytic effect of calcium and potassium on CO_2_ gasification of Shengli lignite: the role of carboxyl**

**Yanpeng Ban, Yan Wang, Na Li, Runxia He, Keduan Zhi*, Quansheng Liu***

*College of Chemical Engineering,* *Inner Mongolia University of Technology,* *Inner Mongolia Key Laboratory of High-Value Functional Utilization of Low Rank Carbon Resources, Huhhot 010051,* *Inner Mongolia, China*

*Corresponding author.

E-mail address: *liuqs@imut.edu.cn (Q. Liu)* *zhikeduan@gmail.com.(K. Zhi).* Tel.0086+13664740405.

**Repeatability and reproducibility**

Fig.1 Repeatability and reproducibility of coal gasification

**Raman data processing**

In order to get more detailed carbon skeleton structure of coal, each sample was fitted into five peaks according to literatures. [1] Each spectrum was subjected to peak fitting using a curve fitting software, Origin8.5/Peak Fitting Module, to resolve curve into 4 Lorentzian bands and 1 Gaussian band (designated for the D3 band). The parameters including peak position, full width at half maximum (FWHM), intensity, and integrated area of each band were derived from the decomposition. The band area ratios I_G_/I_All_ and I_D1_/I_G_ were chosen, because both were found to well represent the char microstructure order. The microcrystalline planar crystalline size L_a_ for the samples was calculated from the integrated intensities of the D_1_ and G bands on the Raman spectra using equation 1.

$$L_{a}=C\left( \lambda_{L} \right)\left[ {I_{D1}}/{I_{G}} \right]^{-1}$$

where C(λ_L_) is the wavelength pre-factor and I_D1_ and I_G_ are the area of the D_1_ and G bands respectively. Mattews et al. [2] considered a wavelength dependency of C and presented the following relation:

$$C\left( \lambda_{L} \right)\approx C_{0}+\lambda_{L}C_{1}$$

where C_0_=12.6 nm and C_1_=0.033 . λ_L_=532nm.

**XPS data processing**

The calibration of the main C 1s peak was carried out at approximately 284.6eV. Data processing was performed by XPS peak separation software (XPS PEAK4.1). It is well established in aliphatic chemistry that binding energy shifts which occur in the Cls peak due to the presence of carbon-oxygen functional groups, it can be classified in the following manner; C-C and C-H hydrocarbon (aromatic or aliphatic) at (284.6±0.3) eV; C-O, ether or hydroxyl at (286.3±0.2)eV; C=O, carbonyl at (287.5±0.3)eV and-COO, carboxyl at (289.0±0.1)eV.[3] Using XPS peak fit analysis technology; the contents of these four groups could be calculated.

[1] Sadezky A, Muckenhuber H, Grothe H, et al. 2005 Raman microspectroscopy of soot and related carbonaceous materials: spectral analysis and structural information[J]. Carbon, 43(8): 1731-1742.(doi.org/10.1016/j.carbon.2005. 02.018)

[2] Matthews M J, Pimenta M A, Dresselhaus G, et al. 1999 Origin of dispersive effects of the Raman D band in carbon materials[J]. Physical Review B, 59(10): R6585. (doi:org/10.1103/PhysRevB.59.R6585)

[3] Ban Y, Tang Y, Wang J, et al. 2016 Effect of inorganic acid elution on microcrystalline structure and spontaneous combustion tendency of Shengli lignite[J]. J. Fuel Chem. Technol., 44(9): 1059-1065.(doi:org/10.1016/S1872-5813(16) 30047-0)
